# Public trust in physicians: empirical analysis of patient-related factors affecting trust in physicians in China

**DOI:** 10.1186/s12875-022-01832-6

**Published:** 2022-08-30

**Authors:** Changle Li, M. Mahmud Khan

**Affiliations:** 1 Department of Health Economics, School of Health Management, Inner Mongolia Medical University, Hohhot, China; 2 Department of Health Policy and Management, College of Public Health, University of Georgia, 100 Foster Rd, Wright Hall 116, Athens, GA 30602 USA

**Keywords:** Public trust in physicians, Patient-physician relationship patient-related factors, China

## Abstract

**Background:**

Trust between the parties is essential for the efficient functioning of the healthcare market. Physician-patient relationship represents an asymmetric information situation and trust in physicians is critical for improving health and wellbeing of patients. In China, trust in physicians appears to be quite low creating conflicts between physicians and patients. This study aims to identify some general factors associated with trust in physicians in general using a nationally representative survey.

**Methods:**

A cross-sectional analysis using data from 2018 China Family Panel Study (CFPS). Survey responses of individuals aged 16 years or above were extracted from CFPS and the final sample consisted of 29,192 individuals. An ordered probit model was used to identify factors causing heterogeneity in the levels of trust in physicians.

**Results:**

Higher educational attainment and having medical insurance coverage are associated with higher likelihood of trusting physicians. Older adults (> = 30 years), males, urban residents, wage-earners, and self-employed persons are less likely to trust physicians. People who are diagnosed as chronic diseases or current smokers indicate lower level of trust in physicians. Higher perceived quality of services improves trust.

**Conclusion:**

Socioeconomically disadvantaged population groups and uninsured individuals are less likely to trust physicians. Health care delivery system needs to address the concerns of these specific population groups to reduce tensions between physicians and patients. Increasing health insurance coverage and offering insurance with low out-of-pocket expenses should reduce the perception that physicians are more guided by their income rather than the wellbeing of patients. The system should also develop a comprehensive bill of rights of patients to improve patient-physician relationship.

**Supplementary Information:**

The online version contains supplementary material available at 10.1186/s12875-022-01832-6.

## Background

In presence of asymmetric information, trust between the transacting parties is essential for efficient functioning of the market. Physician-patient relationship is characterized by this type of asymmetric information situation and trust becomes a critically component of service provision and its effectiveness [1]. The degree of trust patients has in their physicians is an important aspect to consider for reforming health system and developing insurance programs and policies.

Patients’ lack of trust in physicians lowers quality of care, discourage use of preventive services and adversely affect patients’ adherence to clinical advice. High degree of distrust can even lead to violence and destruction of medical equipment and properties unless the legal system allows easy remedies for perceived harms created by health care providers. The World Medical Association in its 2020 assembly included an agenda item that deals with “surge of violence against health personnel worldwide” [2]. A news item published in the Guardian in 2007 reported that 5500 medical workers were injured in China in 2006 in assaults, causing more than 200 million yuan in damages to medical equipment and infrastructure [3]. One article in 2014 lists 17 violent encounters reported in news media in just 1 month in 2014 [4]. The COVID-19 has probably made the situation even worse for health care providers around the world. The principal reason for the violence against health care providers is “patient perception of injustice within the medical sphere, related to profit mongering, knowledge imbalances and physician conflict of interest” [5].

Despite the importance of “trust” in health care market, there is no standardized empirical measure of degree of trust and many alternative measures have been proposed. The definition of trust in physicians often means acceptance of the idea that physicians act in the best interest of patients when recommending treatment and medical management of diseases and not by their short-term economic and social interests [1, 6–8]. Published literature identifies two forms of trust in physicians: interpersonal trust and public trust [9]. Interpersonal trust refers to trust between a patient and his/her health care provider. Public trust means trust in generalized collective entities, i.e., physicians in general, not a specific physician with whom the patient has interacted with in the recent past [7, 9]. Public trust and interpersonal trust are, however, related and high degree of interpersonal trust often implies high degree of public trust. Public trust is a reflection of comprehensive opinion, in part influenced by patients’ personal experiences with their own health care providers and partly influenced by the image of physicians through social media and mainstream media or other social avenues [9, 10]. A variety of respondents who are even without identified physicians within established treatment relationships can assess public trust in physicians. Therefore, public trust could be an indicator to measure the performance of healthcare system [9]. In this study, the focus is on the public or generalized trust of clients in physicians as a group.

Several tools are being used to measure the level of interpersonal trust between physicians and patients [6, 11–13]. However, much less effort has been made to measure public trust. Hall et al. [9] and Dugan et al. [14] developed and tested an 11-item and a 5-item measure for public trust based on a multidimensional conceptual framework. Cronbach’s alpha was 0.89 for the 11-item measure and 0.77 for the 5-item measure, respectively [9. 14]. On the other hand, since the idea of public trust in physicians (or any other health care providers) wants to measure the degree of trust consumers have in physicians in general, empirical measurement of generalized trust often uses one or two simple questions in surveys. For example, Huang et al. [15], Zhao & Zhang [16], and Yuan & Lee [17] used a 5-point Likert scale item to measure public trust based on a question (“All things considered, doctors in your country can be trusted”) from the International Social Survey Program. Moreover, Chen et al. [18] employed two 7-point Likert scale questions (“Overall, I trust the physicians” and “Most physicians are trustworthy”) to measure public trust. Moreover, the single-item response scale is literally and conceptually compatible with a validated multi-item measure for public trust proposed by Hall et al. [9] and Dugan et al. [14].

Review of literature imply that a host of factors affect trust in physicians. The consumer characteristics affecting trust in physicians include gender, age, race, educational attainment, marital status, occupation, place of residence, self-rated health status, medical condition, health-related behavior, medical insurance coverage, income, satisfaction with medical care received, and clinical experience [16, 19–27]. Physician characteristics are also found to be associated with “trust” and these include physician age, gender, body mass index, practice location, specialty, physician emotional intelligence, and physician behavior [28–32]. The physician-patient interactions define not only the trust in physicians but also the quality of physician-patient relationship and continuity of the relationship [12, 29, 33, 34].

The deterioration of physician-patient relationship has become a severe social problem and concern in China. Physician-patient conflict exacerbated in recent years due to lack of trust in physicians in general and physician recommendations or advice in particular [35]. Several studies have examined trust in physicians and its determinants in China. Some analyzed the determinants of interpersonal trust in physicians using samples derived from public hospitals in different provinces and municipalities [24, 26, 36]. However, only one article employs the International Social Survey Program data and telephone interview to examine public trust in physicians [16]. Another article discussed the relationship between health information acquisition and interpersonal trust in physicians in Beijing and Hefei [37].

Most studies in China have focused on the interpersonal trust, not how the consumers view physicians in general. Unlike many developed countries, the concept of ‘my physician’ is not common in China, mainly due to lack of family physicians in the primary care system and high degree of bypassing of primary care providers to obtain care from upper level facilities [38]. In addition, most of the studies on China did not use nationally representative data set, and, therefore, the results may not be generalizable. To fill these research gaps, this study intends to identify the factors associated with trust in physicians using a nationally representative survey.

## Methods

### Data

China Family Panel Studies (CFPS), conducted by the Institute of Social Science Survey of Peking University, is the data set used for this analysis. The sample of CFPS was drawn from 25 provinces and their administrative equivalents. The population of 25 provinces represents 95% of total population of Mainland China. A multistage probability sample proportional to size was used for the survey. More details on the sampling procedure and data collection process are available in Xie and Hu [39]. The CFPS is a nationally representative longitudinal survey that collects information by using community, family, adult, and child questionnaires. The survey consists of the following modules: demographics, family structure/transfer, health status and functioning, biomarkers, health care and insurance, work, income and consumption, assets (individual and household), and community-level information.

The CFPS respondents are reinterviewed every 2 years, with the first wave in 2010 and four follow-ups in 2012, 2014, 2016, and 2018. The 2018 survey included 30,593 adults (aged ≥16 years) who answered the survey questionnaire. After eliminating all cases with missing relevant data, the final sample consisted of a total of 29,166 adults.

### Measures

#### Dependent variable

Public trust in physicians was set as an ordinal dependent variable. In the CFPS, each adult was asked, ‘Considering all things together, how much do you trust physicians in China? Please answer by picking a number in between 0 and 10, where 0 stands for not at all and 10 for completely (11-point Likert scale)’. The use of 11-point Likert scale as it increases sensitivity and reduces skewness compared to the 4-,5-, and 6-point Likert scale [40].

#### Independent variables

Based on apriori considerations, several factors may affect public trust in physicians. The analysis considered the following six categories of variables to explain the degree of trust in physicians: (1) socio-demographic characteristics (age, gender, educational attainment, marital status, place of residence, medical insurance coverage, household income, employment status, location, and family size), (2) health status (self-rated health status and chronic conditions), (3) health-related behaviors (current smoking and regular drinking), (4) past clinical experiences (hospitalization and perceived quality of physician services), (5) Channels of information acquisition (Internet and television), and (6) health system performance (the total health expenditures as a percentage of gross domestic product and the government’s share of total health spending). Definitions of all the relevant variables are listed in Table 1.

**Table 1:** Definitions of variables used in the empirical analysis of trust in physicians (China Family Panel Study 2018)

Variable	Description	Percent/mean
**Dependent variable**		
*Public trust in physicians*	An ordinal variable with 11 response categories from 0 (completely distrust) to 10 (completely trust)	6.74^a^
**Independent variable**		
*Overall patient satisfaction*		
Very Unsatisfied	1 if the individual is very unsatisfied with the condition of health facility he/she often visits; 0 otherwise	1.51
Unsatisfied	1 if the individual is unsatisfied with the condition of health facility he/she often visits; 0 otherwise	8.48
Fair	1 if the individual is ok with the condition of health facility he/she often visits; 0 otherwise	22.88
Satisfied	1 if the individual is satisfied with the condition of health facility he/she often visits; 0 otherwise	59.40
Very Satisfied	1 if the individual is very satisfied with the condition of health facility he/she often visits; 0 otherwise	7.73
*Patient evaluation of medical expertise and knowledge (physician competency)*		
Very Bad	1 if the individual evaluates medical competency of physician often visited as very bad; 0 otherwise	1.82
Bad	1 if the individual evaluates medical competency of physician often visited as bad; 0 otherwise	10.28
Fair	1 if the individual evaluates medical competency of physician often visited as fair; 0 otherwise	33.49
Good	1 if the individual evaluates medical competency of physician often visited as good; 0 otherwise	44.33
Very Good	1 if the individual evaluates medical competency of physician often visited as very good; 0 otherwise	10.07
*Age (years)*		
16–29	1 if the individual is aged 16–29 years; 0 otherwise	19.69
30–39	1 if the individual is aged 30–39 years; 0 otherwise	16.77
40–49	1 if the individual is aged 40–49 years; 0 otherwise	18.02
50–59	1 if the individual is aged 50–59 years; 0 otherwise	19.72
> =60	1 if the individual is aged > = 60 years; 0 otherwise	25.80
*Gender*	1 if the individual is male; 0 for female	49.74
*Educational attainment*		
Illiterate/Semi-literate	1 if the individual is illiterate or semi-literate; 0 otherwise	21.66
Elementary school	1 if the individual attended elementary school; 0 otherwise	19.79
Middle school	1 if the individual graduated from middle school; 0 otherwise	30.00
High school	1 if the individual graduated from high school; 0 otherwise	16.28
> 3-years of college	1 if the individual had above three-years of college; 0 otherwise	12.26
*Married*	1 if the individual is married; 0 otherwise	78.71
*Place of residence*	1 if urban resident; 0 for rural resident	50.70
*Medical insurance*		
GMI	1 if enrolled in Government Medical Insurance; 0 otherwise	2.37
UEMI	1 if enrolled in Urban Employee Medical Insurance; 0 otherwise	14.59
URMI	1 if enrolled in Urban Resident Medical Insurance; 0 otherwise	8.51
NRCMI	1 if enrolled in New Rural Cooperative Medical Insurance; 0 otherwise	65.72
Sup Insurance	1 if enrolled in supplementary medical insurance; 0 otherwise	0.45
No Insurance	1 if the individual does not have medical insurance; 0 otherwise	8.36
*Household income*		
Low income	1 if household income is in the first quartile; 0 otherwise	24.97
Lower middle income	1 if household income is in second quartile; 0 otherwise	25.09
Upper middle income	1 if household income is in the third quartile; 0 otherwise	25.05
High income	1 if household income is in the highest quartile; 0 otherwise	24.89
*Employment status*		
Agricultural worker	1 if the individual is involved with agricultural jobs; 0 otherwise	31.96
Wage-earner	1 if the individual reports working as wage earner; 0 otherwise	34.20
Self-employed	1 if the individual reports being self-employed rather than working for an employer; 0 otherwise	8.31
Economically inactive	1 if the individual reports being temporary worker, retirement, unemployment, or student; 0 otherwise	25.53
*Self-rated health status*		
Poor	1 if the individual reports health status to be poor; 0 otherwise	16.22
Fair	1 if the individual reports health status to be fair; 0 otherwise	12.97
Good	1 if the individual reports health status to be excellent, very good, or good; 0 otherwise	70.81
*Chronic conditions*	1 if the individual has had doctor-diagnosed chronic diseases in the past six months; 0 otherwise	35.03
*Current smoking*	1 if the individual who currently smokes tobacco products; 0 otherwise	29.05
*Regular drinking*	1 if the individual drinks alcohol at least 3 times a week in past month; 0 otherwise	15.05
*Hospitalization*	1 if the individual reports hospitalization in the past 12 months; 0 otherwise	13.01
*Family size*	Number of members with the household	4.21^a^
*Internet*	1 if the individual reports Internet as a channel of information acquisition to be very important; 0 otherwise	42.28
*Television*	1 if the individual reports television as a channel of information acquisition to be very important; 0 otherwise	40.78
*Locations of respondents*		
Northeast region	1 if the individual lives in the Northeast region; 0 otherwise	13.25
East region	1 if the individual lives in the East region; 0 otherwise	32.51
Central region	1 if the individual lives in the Central region; 0 otherwise	23.67
West region	1 if the individual lives in the West region; 0 otherwise	30.57
*GDP*^b^	The total health expenditures as a percentage of gross domestic product (GDP)	7.40
*Government spending*^b^	The government’s share of total health spending	28.48 ^a^

^a^Values are expressed as mean

^b^The total health expenditures as a percentage of GDP and the government’s share of total health spending in each province and municipality were collected from the China Health Statistical Yearbooks

This study measured perceived quality of care from two perspectives: provider structural quality and provider competency [41]. The variable, provider structural quality, was grouped into five levels: very unsatisfied, unsatisfied, fair, satisfied, and very satisfied. The question in the CFPS that collected information on structural quality is: ‘Are you satisfied with the condition of the health care facility that you visit most often (such as the adequacy of facilities, equipment, staff, and drug, qualifications of physicians and nurses, and administrative structures)?’ Another variable, provider competency, was also divided into five categories: very bad, bad, fair, good, and very good, based on a question that asks: ‘How would you evaluate the knowledge, expertise, skills, and abilities of the health care provider that you visit most often?’

### Statistical analysis

To discuss the level of trust reported by individuals, a descriptive analysis of trust has been presented by considering various individual characteristics and experiences. Since the survey uses a complex sample design, it is important to include weights in the analysis to reflect population level estimates. For descriptive analysis, we have categorized reported levels of public trust in physicians into three groups: low-level of trust (0–3), medium-level of trust (4–6), and high-level of trust (7–10). The purpose of the descriptive analysis is to help identification of potentially relevant variables affecting level of trust. Pearson’s chi-square test was performed for univariable analysis.

For formal empirical modeling of public trust in physicians, we have employed ordered logistic model to identify factors affecting the level of trust in physicians among Chinese adults. This model is based on a latent regression and is defined as follows:

$${y}^{\ast }={x}^{\prime }a+\varepsilon$$

*x*^′^ is a vector of independent variables identified based on literature review and the descriptive analysis of level of trust in physicians. In the equation, *a* is the coefficient vector. *y*^∗^ is an unobserved latent variable linked to the observed ordinal response categories related to “trust in physicians” (*TP*). The errors *ε* are normally distributed across observations and standardized at mean of zero and variance of 1.

$$TP=\left[\begin{array}{c}\begin{array}{cc}\ 0,&\ if\ {y}^{\ast}\le {\mu}_0\ \\ {}\ 1,&\ if\ {\mu}_0<{y}^{\ast}\le {\mu}_1\end{array}\\ {}.\\ {}.\\ {}.\\ {}\ 9, if\ {\mu}_8<{y}^{\ast}\le {\mu}_9\\ {}10, if\ {\mu}_9<{y}^{\ast}\\ {}\ \end{array}\right]$$

where *μ* are the underlying thresholds that defines theoretical distribution of level of trust, subject to the constraint that 0 < *μ*_1_ < *μ*_2_ < ⋯ < *μ*_9_. The ordered logistic model relies on the parallel-lines assumption, which means the coefficient vector *a* is identical for all categories of *TP*. The probability of observing a specific level of trust in physicians can be written as:

$${\displaystyle \begin{array}{c} Prob\ \left( TP=0|x\right)=\varPhi \left(-{x}^{\prime}\beta \right)\\ {} Prob\left( TP=1|x\right)=\varPhi \left({\mu}_1-{x}^{\prime}\beta \right)-\varPhi \left(-{x}^{\prime}\beta \right)\ \\ {}\begin{array}{c}\vdots \\ {} Prob\left( TP=10|x\right)=1-\varPhi \left({\mu}_9-{x}^{\prime}\beta \right)\ \end{array}\end{array}}$$

The ordered probit model was estimated employing maximum likelihood estimation in the statistical software package STATA 17 [42, 43].

## Results

Table 1 shows the characteristics of the study sample. The sample size was 29,166 with 49.7% of respondents being male and 25.8% were of age 60 years or more. About 51% of respondents reported living in urban areas. 58.5% of the respondents completed at least middle school education. Most respondents (91.6%) were enrolled in medical insurance schemes. Table 2 reports individual characteristics by level of trust (low-level, medium-level and high-level of trust). The information in the table indicates that “hospitalization” experience and household income did not show significant relationship with reported levels of trust. Other variables, however, appear to be related with the trust variable. These include provider structural quality, perceived provider competency, age, gender, educational attainment, marital status, place of residence, locations of respondents, medical insurance coverage, employment status, self-rated health status, chronic conditions, channels of information acquisition, and health-related behaviors.

**Table 2:** Relationship between reported trust levels in physicians and individual characteristics, China family panel study 2018, weighted data

	Low-level of trust	Medium-level of trust	High-level of trust	
Provider structural quality (%)				*p* < 0.001
Very Unsatisfied	37.08	34.20	28.72	
Unsatisfied	23.33	41.45	35.22	
Fair	11.91	41.23	46.86	
Satisfied	6.71	31.96	61.34	
Very Satisfied	6.14	22.69	71.17	
Perceived provider competency (%)				*p* < 0.001
Very Bad	33.46	38.91	27.63	
Bad	21.17	39.29	39.55	
Fair	10.81	39.95	49.25	
Good	6.16	30.28	63.55	
Very Good	6.07	26.72	67.21	
Age group (%)				*p* < 0.001
16–29	6.10	30.05	63.85	
30–39	11.46	37.54	50.99	
40–49	11.89	34.45	53.65	
50–59	11.24	36.89	51.87	
> =60	9.00	33.47	57.53	
Gender (%)				*p* < 0.001
Male	11.71	34.14	54.15	
Female	7.87	34.50	57.63	
Educational attainment (%)				*p* < 0.001
Illiterate/Semi-literate	9.98	33.85	56.18	
Elementary school	10.64	34.54	54.82	
Middle school	11.14	34.73	54.13	
High school	9.00	34.21	56.79	
Above three-years of college	7.17	33.91	58.92	
Marital status (%)				*p* < 0.001
Married	10.63	35.26	54.11	
Other	7.64	31.63	60.74	
Place of residence (%)				*p* < 0.001
Urban residents	10.35	35.92	53.73	
Rural residents	9.06	31.80	59.14	
Medical insurance coverage (%)				*p* < 0.05
GMI	9.24	38.78	51.98	
UEMI	10.34	34.92	54.73	
URMI	9.22	37.15	53.63	
NRCMI	9.51	33.33	57.17	
Sup Insurance	11.81	19.17	69.02	
No Insurance	12.02	36.38	51.60	
Household income (%)				*p* = 0.125
Low income	10.06	32.81	57.13	
Lower middle income	10.39	34.32	55.29	
Upper middle income	9.58	33.68	56.74	
High income	9.42	36.29	54.30	
Employment status (%)				*p* < 0.001
Agricultural worker	9.34	32.48	58.19	
Wage-earner	10.20	36.22	53.58	
Self-employed	13.91	36.46	49.63	
Economically inactive	8.46	32.74	58.80	
Self-rated health status (%)				*p* < 0.001
Poor	12.68	34.87	52.45	
Fair	11.25	38.26	50.49	
Good	9.02	33.50	57.48	
Chronic conditions (%)				*p* < 0.05
Yes	10.90	34.97	54.13	
No	9.33	33.99	56.68	
Current smoking (%)				*p* < 0.001
Yes	13.92	34.72	51.36	
No	8.19	34.15	57.66	
Regular drinking (%)				*p* < 0.01
Yes	11.56	35.50	52.94	
No	9.50	34.07	56.43	
Hospitalization (%)				*p* = 0.304
Yes	9.59	32.96	57.45	
No	9.88	34.50	55.61	
Internet (%)				*p* < 0.01
Very important	8.51	32.94	58.54	
Otherwise	10.97	35.47	53.56	
Television (%)				*p* < 0.01
Very important	8.57	31.17	60.26	
Otherwise	10.67	36.36	52.97	
Locations of respondents (%)				*p* < 0.01
Northeast region	17.8	33.88	48.32	
East region	8.01	34.11	57.88	
Central region	9.60	35.90	54.50	
West region	8.99	33.38	57.64	

**Fig. 1:**
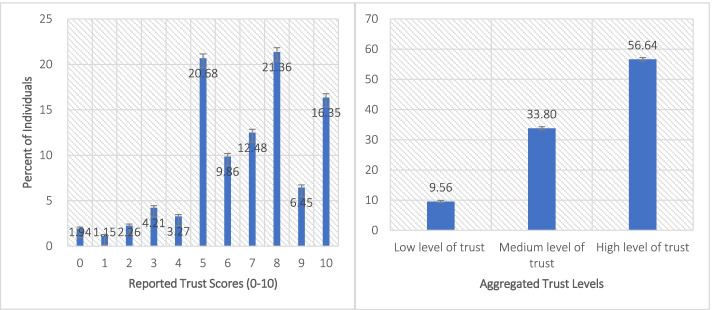
Percent of individuals reporting trust in physicians by levels (0–10 scale of trust level and three aggregated levels)

Figure 1 shows the distribution of public trust in physicians on the 0–10 scale as well as on three derived levels of trust using the individual responses on 0–10 scale. The plot of reported values for the 0–10 trust scale shows that the distribution is skewed to the left with most respondents reporting trust in the range “5” to “10”. It also shows some lumping of values at 5, 8 and 10 implying some errors in reporting the level of trust. Figure 1 also shows 95% confidence intervals for the each reported trust score and the aggregated three levels. About 57% of individuals reported high level of trust in physicians (95% CI:56.1, 57.2%). Only 9.6% (95% CI: 9.2, 9.9%) reported low-level of trust in physicians.

The results of the ordered probit regression analysis are reported in Table 3. The results imply that people aged 30 years or older were less likely to trust physicians compared to people in the age group 16–29 years (Coef. = − 0.077, *p* < 0.01; Coef. = − 0.050, *p* < 0.05; Coef. = − 0.087, *p* < 0.01). Female respondents are more likely to trust physicians than the males (Coef. = − 0.065, *p* < 0.01). Probability of trust in physicians increased with higher educational attainment. For example, attending elementary school, completion of middle school, and completion of high school were associate with lower trust in physicians compared to those who had above three-years of college (Coef. = − 0.078, *p* < 0.01; Coef. = − 0.093, *p* < 0.01; Coef. = − 0.099, *p* < 0.01). Married people were less likely to trust physicians than those who were never married, separated, divorced, or widowed (Coef. = − 0.038, *p* < 0.05). Urban residents reported lower likelihood of trusting physicians compared to rural residents (Coef. = − 0.110, *p* < 0.01). People who were enrolled in Urban Employee Medical Insurance and New Rural Cooperative Medical Insurance were more likely to trust physicians than those who were uninsured (Coef. = 0.065, *p* < 0.05; Coef. = 0.114, *p* < 0.01). Agricultural workers had an increased likelihood of trusting physicians compared to those who were economically inactive (Coef. = 0.058, *p* < 0.01). Wage-earners and self-employed individuals reported lower trust in physicians compared to those who were economically inactive (Coef. = − 0.073, *p* < 0.01; Coef. = − 0.126, *p* < 0.01).

**Table 3:** Trust in Physicians and Individual Characteristics Affecting the Level of Trust: Results of Ordered Probit Regression Model (Household Level)

	Coef.	SE
**Provider/facility structural quality**		
Very Unsatisfied (ref.)		
Unsatisfied	0.169^**^	0.067
Fair	0.360^***^	0.066
Satisfied	0.557^***^	0.065
Very Satisfied	0.855^***^	0.072
**Perceived medical competency of physician**		
Very Bad (ref.)		
Bad	0.124^**^	0.058
Fair	0.284^***^	0.057
Good	0.460^***^	0.057
Very Good	0.618^***^	0.061
**Age group**		
16–29 (ref.)		
30–39	−0.077^***^	0.022
40–49	−0.050^**^	0.024
50–59	− 0.087^***^	0.026
> =60	−0.033	0.028
**Respondent male**		
Yes	−0.065^***^	0.015
**Educational attainment**		
Illiterate/Semi-literate	−0.295	0.030
Elementary school	−0.078^***^	0.027
Middle school	−0.093^***^	0.022
High school	−0.099^***^	0.022
Above three-years of college (ref.)		
**Respondent married**		
Yes	−0.038**	0.018
**Place of residence: urban**		
Yes	−0.110^***^	0.016
**Medical insurance coverage**		
GMI	0.006	0.042
UEMI	0.065^**^	0.029
URMI	0.042	0.030
NRCMI	0.114^***^	0.025
Sup Insurance	0.142	0.092
No Insurance (ref.)		
**Household income**		
Low income (ref.)	−0.009	0.021
Lower middle income	−0.041	0.022
Upper middle income	−0.046	0.024
High income		
**Employment status**		
Agricultural worker	0.058^***^	0.020
Wage-earner	−0.073^***^	0.018
Self-employed	−0.126^***^	0.025
Economically inactive (ref.)		
**Self-rated health status**		
Poor (ref.)		
Fair	0.037	0.025
Good	0.054^**^	0.021
**Chronic condition present**		
Yes	−0.040^***^	0.015
**Current smoker**		
Yes	−0.041^***^	0.017
**Regular drinker**		
Yes	−0.026	0.019
**Hospitalization history**		
Yes	0.035	0.020
**Family size**	0.138^***^	0.004
**Internet very important source of information**		
Yes	0.105^***^	0.015
**Television very important source of information**		
Yes	0.213^***^	0.014
**Geographic Locations of respondents**		
Northeast region (ref.)		
East region	0.186^***^	0.029
Central region	0.164^***^	0.035
West region	0.148^***^	0.035
**GDP**	0.001	0.002
**Government spending**	0.004	0.006

Asterisks^***^ indicates statistical significance at the 1% level, ^**^ at the 5% level

Self-reported good health status increased the likelihood of trusting physicians (Coef. = 0.054, *p* < 0.05). Individuals with chronic health conditions were less likely to trust physicians (Coef. = − 0.040, *p* < 0.01). Those who currently smoke tobacco products reported decreased odds of trusting physicians (Coef. = − 0.041, *p* < 0.01). Provider structural quality and provider competency were positively associated with trusting physicians. Individuals who reported being satisfied with the structural quality of health facility (such as the adequacy of facilities, equipment, staff, and drug, qualifications of physicians and nurses, and administrative structures) were more likely to report high degree of trust in physicians (Coef. = 0.169, *p* < 0.05; Coef. = 0.360, *p* < 0.01; Coef. = 0.557, *p* < 0.01; Coef. = 0.855, *p* < 0.01). Perceived medical competency of physician were positively associated with trust in physicians. Survey respondents who evaluated the medical expertise and knowledge of healthcare providers as high were more likely to trust physicians (Coef. = 0.124, *p* < 0.05; Coef. = 0.284, *p* < 0.01; Coef. = 0.460, *p* < 0.01; Coef. = 0.618, *p* < 0.01).

The probability of trusting physicians increases with a bigger family size (Coef. = 0.138, *p* < 0.01). Individuals who lived in the East region, the Central region, and the West region were more likely to trust physicians compared to those who lived in the Northeast region (Coef. = 0.186, *p* < 0.01; Coef. = 0.164, *p* < 0.01; Coef. = 0.148, *p* < 0.01). People who reported the Internet as a very important channel of information acquisition had a higher odds of trusting physicians (Coef. = 0.105, *p* < 0.01), and the same was true for television as a critical channel of information acquisition (Coef. = 0.213, *p* < 0.01).

## Discussion

The principal objective of the study was to identify the factors associated with public trust in physicians among individuals 16 years of age or older in China using nationally representative survey data. Two-fifths of individuals surveyed reported low or medium level of trust in physicians, which is consistent with the findings in countries like Poland, Chile, and the USA. There are some countries, however, where public trust in physicians is quite high (such as Switzerland, Belgium, and Denmark) [44]. The trust in physicians across countries may have been influenced by health system related factors like health insurance coverage rate, levels of out-of-pocket payments, degrees of access to care, method of paying or reimbursing physicians as well as various individual characteristics including underlying prevalence of diseases and medical conditions. Since the individuals with lower trust in physicians are less likely to accept physician recommendations and more likely to have disputes with their health care providers [45], addressing the trust issue should have significant impact on quality of care and health outcomes of patients. Therefore, identifying the characteristics of individuals with lower trust in physicians will be of interest to policymakers.

We have used the ordered probit regression model to identify the factors affecting trust in physicians in general. The results indicate that age of the person was significantly correlated with trust in physicians. Relatively older individuals (> = 30 years) show a lower probability of trusting physicians. This result is not consistent with the findings reported for the United Kingdom (UK) and the United States (US) [33, 46]. It is possible that the trust in physicians is affected by the age difference between the patient and the physician [34]. In China, primary care physicians’ median age was 39 years in urban areas and 47 years in rural areas [45]. Older patients may view the primary care providers as too young and inexperienced and this perception may lower the probability of trusting physicians. On the other hand, in the UK and US, a high proportion of patients have had their regular physician. Older people are more likely to interact with their regular physicians because of chronic health conditions and have more time to establish a trusting relationship with their regular physicians [47]. However, a low proportion of Chinses patients have had their regular physicians due to a lack of family physicians or general practitioners in the primary care system [38].

We found that men reported lower probability of trusting physicians compared to women. In general, women tend to use more healthcare services than men and higher utilization probably helps in finding a good match between the patient and the physician. In general, trust in physicians is positively associated with the frequency of physician visits, although higher visits may also be viewed as an outcome of mutual trust between patients and physicians [48].

Individuals with a higher level of educational attainment showed increased odds of trusting physicians. Educational attainment is an important indicator of socioeconomic status. Higher socioeconomic status and better knowledge about the health care system indicate higher skills in managing physician-patient communication, and higher financial ability to select the physicians they can trust. Married people show a lower probability of trusting physicians. Unmarried, separated, or divorced people preferred involvement in medical decision-making more than married people [49]. People involved in medical decision-making can create effective doctor-patient communication and have a higher probability of trusting physicians [50]. Family size was positively associated with trusting physicians. The bigger family size increased the probability of satisfaction with their family members’ healthcare. Patients satisfied with their family members’ healthcare had a higher probability of trusting physicians [51].

We found that people who were enrolled in basic medical insurance schemes were more likely to trust physicians compared to the uninsured individuals. China’s medical insurance schemes reduce out-of-pocket expenditure [45] and lower out-of-pocket cost may reduce tensions between the physicians and patients [52]. It is also possible that the insurance system itself is viewed as an independent agency to protect the interests of patients. This would imply that universal health coverage with relatively low out-of-pocket expenses, especially for economically disadvantaged population, will improve trust between patients and physicians and reduce potential conflicts between them. Health system of China needs to improve access to medical care services for socially and economically disadvantaged population groups. China has been successful in expanding health insurance coverage but those remaining uninsured are probably the most vulnerable. Even with insurance, out-of-pocket cost may remain high, especially for individuals with poorer health status. High out-of-pocket expenses and trust in physicians are related and thus, expanding medical insurance to achieve universal coverage with in-built protection from catastrophic health expenditure will reduce patient-physician conflicts significantly. The system should also develop a comprehensive bill of rights of patients to improve patient-physician relationship.

We found that urban residents were less likely to trust physicians than their rural counterparts. The primary health care delivery system in rural China performs better than the system in urban areas [52]. China has invested significant amount of resources to strengthen medical infrastructure in rural areas and patients in rural areas are more likely to receive prompt attention for common medical conditions. Since the physicians in rural areas are not as busy as in urban areas, they can spend more time with patients improving the quality of patient-physician interactions [53]. The results also indicate that wage-earners and self-employed people had a lower probability of trusting physicians compared to individuals not in the labor force. Individuals involved in economically productive activities in China often find it difficult to visit the same physician for their healthcare needs implying lack of continuity of care. Lower continuity of care is related to lower level of trust in physicians [46].

Self-reported good health status increased the probability of trusting physicians. Conversely, individuals with chronic medical conditions and those who smoked at the time of the survey reported lower probability of trusting physicians. Smoking is associated with lower health status and individuals with poorer health are more likely to view their interactions with physicians in a negative manner [25]. If the poor health condition persists, it can also create negative emotions which may adversely affect evaluations of trust in physicians [7].

Higher overall patient satisfaction with medical care services and higher patient evaluation of medical expertise or knowledge of physicians are associated with increased probability of trusting physicians. This is not unexpected – patient satisfaction with medical care and favorable view about physician’s level of expertise and knowledge are the proximate causes of trust in physicians even though both these variables reflect patient experiences with past visits to healthcare providers.

The media used by individuals as the principal source of information appear to affect trust in physicians. The predicted probability of completely trusting physicians is higher when the television is the main media compared to internet as the source of information (see Appendix Table 1). Browsing the network news may be easier to obtain non-neural news about physicians than watching television in China [35]. The results of conditional marginal effects of locations of respondents on complete trust in physicians imply that people in the East and Central part of China trust their physicians more than the people in the West and Northeast regions (see Appendix Table 1). With the weakest economic growth, the Northeast region is known as China’s rustbelt and the region consistently reported lower public satisfaction with the healthcare system. On the other hand, the Central and West regions implemented many healthcare reform policies after piloting in the East region. As a result, the East, Central, and West regions reported significant improvement in public satisfaction with the healthcare system [54].

Several limitations of the study should be noted. One of the important limitations is the way the “trust in physicians” was measured in the survey. The public trust in physicians is a single 11-point Likert scale question and there are no follow-up questions to understand the reasons for selecting a specific “trust level” by the individual. Even when a respondent says that he/she has never seen a physician, the survey still asks the respondent to answer this question. Therefore, considerable errors in reporting may be present in the survey. Second, the CFPS survey does not collect information on the characteristics of the healthcare providers the survey respondents or their family members have used in the recent past. Characteristics of physician and physician’s specialties are likely to be important in affecting the level of trust but it was not possible to incorporate these variables in the model. The data collected on general trust in physicians did not distinguish whether the responses refer to physicians in primary care, secondary care or tertiary care. Third, the data used in this study were collected via survey using a standardized instrument, and thus the limitations of self-reported data such as recall bias and reliability of responses in the presence of interviewers, etc. also applies here. Finally, the study is limited by the information it collected and additional variables that may affect individual’s trust in physicians could not be analyzed. We recommend that further studies be performed to assess the impact of social norms, family doctor system, and payment system reform on general trust in physicians.

## Conclusions

Our analysis found that higher educational attainment and having medical insurance coverage show a higher probability of trusting physicians. Older adults (> = 30 years), urban residents, wage-earners, and self-employed persons are less likely to trust physicians. Individuals who reported their health being poor or those who smoked at the time of the survey had lower probability of trusting physicians. The empirical estimation also found positive effects of higher perceived quality of facility infrastructure and higher medical expertise or knowledge of physicians on trust. Improving trust in physicians is an important policy issue for China because of the increasing incidence of tensions and conflicts between physicians and patients. This analysis has identified several factors and some of these factors are amenable to policy changes.

Rural population reported much higher trust in physicians than the urban residents. This probably implies that investments in primary health care delivery structure with a focus on patients will help improve trust between patients and physicians. In rural areas, the physician care in China is more integrated than in urban areas with relatively high degree of continuity of care. China may consider establishing primary care delivery structure that will allow stronger patient-physician bond. Physician-patient communication is an important aspect of enhancing trust in healthcare providers and training of physicians should include how to be respectful to patients and how to demonstrate sensitivity to patient needs, especially in busy physician practices. Although, this study could not examine the possible negative effects of physician payment system, mechanisms for reducing supplier-induced demand and creating an environment in which health of patients are emphasized rather than volume of medical services and products used should significantly improve mutual trust between patients and physicians.

## Supplementary Information

**Additional file 1 Appendix Table 1.** Estimated conditional marginal effect of specific general factors on complete trust in physicians.

## Data Availability

The dataset used for drafting the paper is a publicly available through the Peking University Open Research Data Platform repository. The dataset is downloadable for research purposes through the link: https://opendata.pku.edu.cn/dataset.xhtml?persistentId=doi:10.18170/DVN/45LCSO.
