# Supplementary material for: Public trust in physicians: empirical analysis of patient-related factors affecting trust in physicians in China
Source: BMC Prim Care. 2022 Aug 30;23:217. doi: 10.1186/s12875-022-01832-6 (PMC9427175; doi:10.1186/s12875-022-01832-6)
Supplement: Supplementary file 1 — Additional file 1 Appendix Table 1. Estimated conditional marginal effect of specific general factors on complete trust in physicians. [file 12875_2022_1832_MOESM1_ESM.docx]

**Appendix**

**Table 1. Estimated conditional marginal effect of specific general factors on complete trust in physicians**

|  | | | | Coef. | | | | | SE | | | |  |
| --- | --- | --- | --- | --- | --- | --- | --- | --- | --- | --- | --- | --- | --- |
| **Provider/facility structural quality** | | | | | | | | | | | | |  |
| Very Unsatisfied (ref.) |  | | | | | | |  | | | |  |  |
| Unsatisfied | 0.024^***^ | | | | | | | 0.008 | | | |  |  |
| Fair | 0.058^***^ | | | | | | | 0.008 | | | |  |  |
| Satisfied | 0.102^***^ | | | | | | | 0.008 | | | |  |  |
| Very Satisfied | 0.186^***^ | | | | | | | 0.012 | | | |  |  |
| **Perceived medical competency of physician** | | | | | | | | | | | | |  |
| Very Bad (ref.) |  | | | | | | |  | | | |  |  |
| Bad | 0.020^**^ | | | | | | | 0.009 | | | |  |  |
| Fair | 0.051^***^ | | | | | | | 0.009 | | | |  |  |
| Good | 0.091^***^ | | | | | | | 0.009 | | | |  |  |
| Very Good | 0.134^***^ | | | | | | | 0.011 | | | |  |  |
| **Age group** | |  | | | | | | |  | | | |  |
| 16-29 (ref.) |  | | | | | | |  | | | |  |  |
| 30-39 | -0.018^***^ | | | | | | | 0.005 | | | |  |  |
| 40-49 | -0.012^**^ | | | | | | | 0.006 | | | |  |  |
| 50-59 | -0.020^***^ | | | | | | | 0.006 | | | |  |  |
| >=60 | -0.008 | | | | | | | 0.007 | | | |  |  |
| **Gender** | |  | | | | | | |  | | | |  |
| Male | -0.015^***^ | | | | | | | 0.003 | | | |  |  |
| **Educational attainment** | |  | | | | | | |  | | | |  |
| Illiterate/Semi-literate | -0.007 | | | | | | | 0.007 | | | |  |  |
| Elementary school | -0.018^***^ | | | | | | | 0.006 | | | |  |  |
| Middle school | -0.022^***^ | | | | | | | 0.005 | | | |  |  |
| High school | -0.023^***^ | | | | | | | 0.005 | | | |  |  |
| Above three-years of college (ref.) | | | | | |  | |  | | | |  |  |
| **Respondent married** | |  | | | | | | |  | | | |  |
| Yes | -0.009** | | | | | | | 0.004 | | | |  |  |
| **Place of residence urban** | |  | | | | | | |  | | | |  |
| Yes | -0.026^***^ | | | | | | | 0.004 | | | |  |  |
| **Medical insurance coverage** | | | | |  | | | |  | | | |  |
| GMI | 0.001 | | | | | | | 0.010 | | | |  |  |
| UEMI | 0.015^**^ | | | | | | | 0.007 | | | |  |  |
| URMI | 0.010 | | | | | | | 0.007 | | | |  |  |
| NRCMI | 0.026^***^ | | | | | | | 0.006 | | | |  |  |
| Sup Insurance | 0.033 | | | | | | | 0.021 | | | |  |  |
| No Insurance (ref.) |  | | | | | | |  | | | |  |  |
| **Household income** | |  | | | | | | |  | | | |  |
| Low income (ref.) | | -0.002 | | | | | | | 0.005 | | | |  |
| Lower middle income | | -0.009 | | | | | | | 0.005 | | | |  |
| Upper middle income | | -0.011 | | | | | | | 0.006 | | | |  |
| High income | |  | | | | | | |  | | | |  |
| **Employment status** | |  | | | | | | |  | | | |  |
| Agricultural worker | 0.014^***^ | | | | | | | 0.005 | | | |  |  |
| Wage-earner | -0.017^***^ | | | | | | | 0.004 | | | |  |  |
| Self-employed | -0.029^***^ | | | | | | | 0.006 | | | |  |  |
| Economically inactive (ref.) | | | |  | | | |  | | | |  |  |
| **Self-rated health status** | |  | | | | | | |  | | | |  |
| Poor (ref.) |  | | | | | | |  | | | |  |  |
| Fair | 0.009 | | | | | | | 0.006 | | | |  |  |
| Good | 0.012^**^ | | | | | | | 0.005 | | | |  |  |
| **Chronic conditions present** | | |  | | | | | |  | | | |  |
| Yes | -0.009^***^ | | | | | | | 0.004 | | | |  |  |
| **Current smoker** | |  | | | | | | |  | | | |  |
| Yes | -0.010^**^ | | | | | | | 0.004 | | | |  |  |
| **Regularly drink** | | | | | |  | | |  | | | |  |
| Yes | -0.006 | | | | | | | 0.004 | | | |  |  |
| **Hospitalized in the past** | |  | | | | | | |  | | | |  |
| Yes | | 0.008 | | | | | | | 0.005 | | | |  |
| **Family size** | | 0.003^***^ | | | | | | | 0.001 | | | |  |
| **Internet as very important information source** | | | | | | | | | |  |  | |  |
| Yes | | 0.024^***^ | | | | | | | 0.004 | | | |  |
| **Television as very important information source** | | | | | | | | | |  | |  |  |
| Yes | | 0.049^***^ | | | | | | | 0.003 | | | |  |
| **Geographic location of respondents** | | | | | | | |  |  | | | |  |
| Northeast region (ref.) | |  | | | | | | |  | | | |  |
| East region | | 0.041^***^ | | | | | | | 0.006 | | | |  |
| Central region | | 0.036^***^ | | | | | | | 0.007 | | | |  |
| West region | | 0.032^***^ | | | | | | | 0.007 | | | |  |
| **GDP** | | 0.001 | | | | | | | 0.001 | | | |  |
| **Government spending** | | 0.001 | | | | | | | 0.001 | | | |  |

Note: Asterisks^***^ indicates statistical significance at the 1% level, ^**^ at the 5% level.
